# Expanding phenotype of MED13-associated syndrome presenting novel *de novo* missense variant in a patient with multiple congenital anomalies

**DOI:** 10.1186/s12920-024-01857-z

**Published:** 2024-05-14

**Authors:** Ekaterina Tolmacheva, Anna S. Bolshakova, Jekaterina Shubina, Margarita S. Rogacheva, Alexey N. Ekimov, Julia L. Podurovskaya, Artem A. Burov, Denis V. Rebrikov, Vladimir G. Bychenko, Dmitry Yu. Trofimov, Gennady T. Sukhikh

**Affiliations:** Kulakov National Medical Research Center for Obstetrics, Gynecology and Perinatology, Moscow, Russia

**Keywords:** Whole exome sequencing, MED13, *De novo* variant, Expanding phenotype

## Abstract

**Background:**

Whole exome sequencing allows rapid identification of causative single nucleotide variants and short insertions/deletions in children with congenital anomalies and/or intellectual disability, which aids in accurate diagnosis, prognosis, appropriate therapeutic interventions, and family counselling. Recently, *de novo* variants in the MED13 gene were described in patients with an intellectual developmental disorder that included global developmental delay, mild congenital heart anomalies, and hearing and vision problems in some patients.

**Results:**

Here we describe an infant who carried a *de novo* p.Pro835Ser missense variant in the MED13 gene, according to whole exome trio sequencing. He presented with congenital heart anomalies, dysmorphic features, hydrocephalic changes, hypoplastic corpus callosum, bilateral optic nerve atrophy, optic chiasm atrophy, brain stem atrophy, and overall a more severe condition compared to previously described patients.

**Conclusions:**

Therefore, we propose to expand the MED13-associated phenotype to include severe complications that could end up with multiple organ failure and neonatal death.

## Background

The incidence of congenital anomaly is estimated to be 2–3% of live births [1]; it is the main cause of neonatal deaths. According to the studies, structural malformations are responsible for about 13% of neonatal intensive care unit admission [2, 3]. Underlying mechanisms include abnormal tissue formation, growth or differentiation influenced by genetic or environmental factors, or a combination of both [4]. About 10–15% of congenital anomaly cases among live born infants are explained by chromosomal aberrations [5]. Regarding diagnostics, chromosomal microarray analysis provides higher resolution for chromosomal rearrangements compared to karyotyping and is currently recommended as a first-tier test for individuals with developmental disabilities or congenital anomalies [6–8]. However, about 40–60% of congenital structural defect causes in neonates remain unsolved [7].

Next generation sequencing technology has dramatically improved our understanding of genetic disease [9–13]. The knowledge about the exact etiology of malformation in an affected infant can change his/her clinical management and potentially impact mortality and morbidity of such patients [14]. Another important possibility provided by a clear genetic diagnosis is proper family counselling and risk assessment regarding the birth of potential siblings.

Whole exome sequencing (WES) covers protein-coding regions throughout the whole genome, including genes not currently associated with monogenic disorders. The diagnostic yield of WES is up to 58% in phenotypically diverse populations [15]. Moreover, WES in the context of trio (proband-parents) sequencing provides faster and easier analysis allowing for segregation analysis (i.e. compound heterozygous and homozygous, *de novo* variants).

Recently, *de novo* variants in the Mediator complex subunit 13 (MED13, OMIM 603,808) were detected in a cohort of 13 patients with intellectual disability and dysmorphisms [16]. Other features that were reported in patients with MED13 variants included optic nerve abnormalities, hearing loss, growth delay/restriction, hypotonia, mild congenital heart anomalies, epilepsy, and microcephaly [17–20].

Here we report a neonate with multiple congenital anomalies, facial dysmorphisms and congenital heart defects, who carries a *de novo* missense variant in MED13, discovered during the Whole-Exome Newborn Screening Project “EXAMEN” (ClinicalTrials.gov Identifier: NCT05325749, performed for all newborns at the Kulakov National Medical Research Center for Obstetrics, Gynecology and Perinatology, Moscow, Russia). The neonate presented with significantly more severe symptoms compared to the previously described in such patients.

## Results

The patient is the third child of a nonconsanguineous healthy 33-year-old, gravida 3 para 3 mother, and 33-year-old father. Family history was unremarkable. During the pregnancy an increased nuchal translucency was revealed, while a normal karyotype was detected using chorionic villous sampling. On the 35th week of gestation large bowel obstruction, cardiac anomaly and growth restriction were noticed on fetal ultrasound examination. The baby was born on the 37th week via vaginal delivery with breech presentation. His birth weight was 2,132 g (2nd centile) and the length parameters were normal (47 cm). The Apgar scores were 7/8. He was immediately placed on non-invasive ventilation and transferred to the neonatal intensive care unit where he was monitored for respiratory insufficiency.

**Fig. 1:**
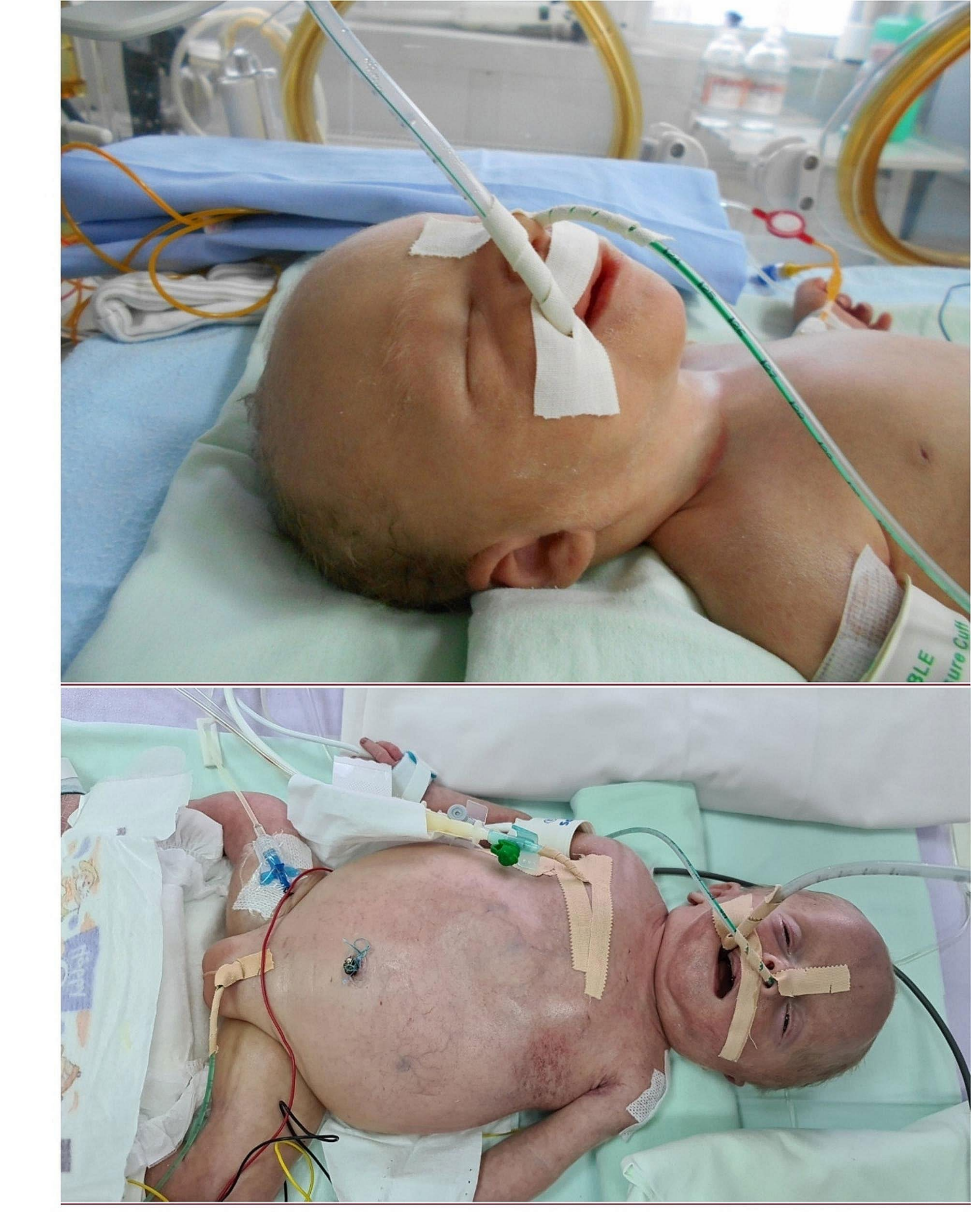
Child on the 1st (upper image) and 34th day of life (lower image). Dysmorphic features including hypertelorism, broad nasal tip, low set simple ears, and wide-spaced nipples can be noted. Parental consent for publishing the photos was obtained

His visual assessment revealed hypertelorism, broad nasal tip, low set simple ears, and wide-spaced nipples (Fig. 1). Ophthalmic examination revealed congenital cataract, optic nerve atrophy, retinal degeneration, and neurogenic lagophthalmos.

An echocardiogram revealed isthmus hypoplasia, ventricular septal defect, small atrial septal defect, right heart hypertrophy, tricuspid insufficiency, and pulmonary hypertension. Chest X-ray demonstrated the symptoms of respiratory distress syndrome. As the patient’s oxygen saturation remained below 90%, he was intubated.

An abdominal ultrasound revealed possible ileal atresia. Neurosonography assessment indicated mild ventriculomegaly.

An open surgery for the resection of the atretic bowel with primary end-to-end anastomosis was performed on the 1st day of life, the baby suffered disseminated intravascular coagulation. On the 10th day the baby was extubated, but subsequently reintubated on the 13th day due to respiratory acidosis. On the 16th day a disseminated intravascular coagulation was suspected according to the clinical symptoms and laboratory findings.

**Fig. 2:**
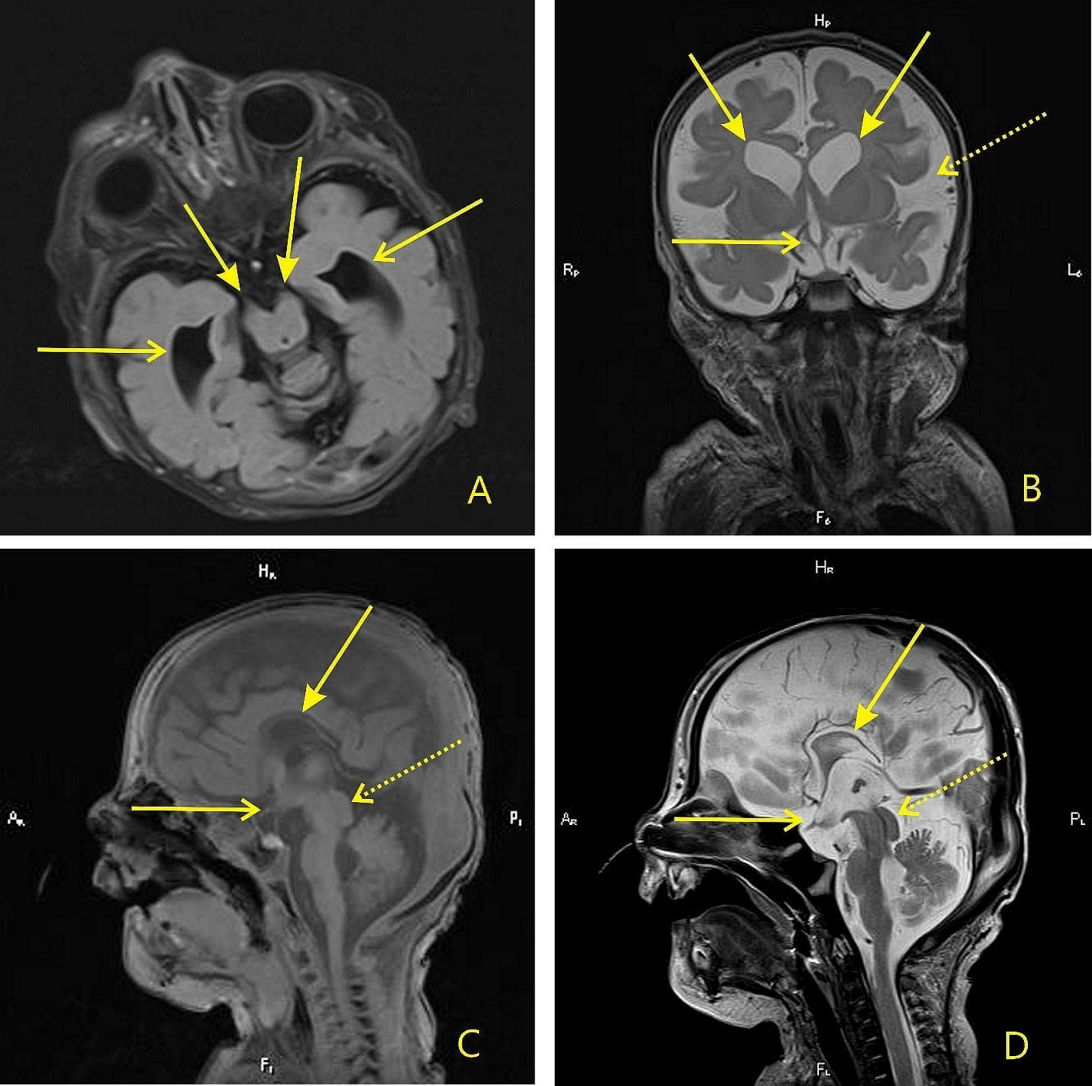
The results of brain MRI performed according to a routine protocol on a Magnetom Verio tomograph with 3T field induction and the use of a neonatal head coil, against the background of controlled sedation under the control of vital functions (SpO2, heart rate). (**A**): FLAIR image in the axial plane, arrows show the cerebral peduncles and brainstem reduced in size, open arrows show the enlarged temporal horns of the lateral ventricles; (**B**): T2WI in the frontal plane, arrows show dilated bodies of the lateral ventricles, the chiasm is hypoplastic (shown by an open arrow), dilated external cerebrospinal fluid spaces (dotted arrow); (**C**): T1WI in the sagittal plane, the arrow shows the hypoplastic corpus callosum, the genu is not developed, the splenium is absent, the hypoplastic chiasm is shown by an open arrow, the enlarged quadrigeminal plate is shown by a dotted arrow. (**D**): T2WI in the sagittal plane, the arrow shows the hypoplastic corpus callosum, the genu is not developed, the splenium is absent, the hypoplastic chiasm is shown by an open arrow, the enlarged quadrigeminal plate is shown by a dotted arrow

Multi-slice spiral computed tomography, performed on the 36th day, revealed bronchopulmonary dysplasia that manifested as respiratory distress. On the 57th day magnetic resonance tomography showed hydrocephalic changes, hypoplastic corpus callosum, bilateral optic nerve atrophy, optic chiasm atrophy, and brain stem atrophy (Fig. 2). The disseminated intravascular coagulation was followed by pulmonary hemorrhage. The baby deceased at 76 days of age due to multiple organ failure. The parents declined autopsy.

## Discussion

*MED13* gene encodes the Mediator complex subunit 13, a part of the Mediator complex that functions with DNA-binding transcription factors and RNA polymerase II for gene activation [21]. Mediator is a large multiprotein complex universally expressed in eukaryotic tissues. It was described as a stabilizing agent of the preinitiation complex, communicating transcription activating signals for the transcription of all protein-coding and many non-coding genes by RNA polymerase II [22]. MED13 is ubiquitous in human tissues, with the highest expression found in the placenta, skeletal muscle, heart, pancreas, and brain [23]. The major role of the complex was indeed proven by the discoveries of multiple patient cases with *de novo* variants in the genes encoding Mediator subunits, such as MED12 [24], CDK8 [25], MED13L [26], and others. The patients carrying *MED13* variants are also suffering neurodevelopmental disorders that are in many cases accompanied by heart anomalies, motor delay, dysmorphic features, microcephaly, deafness, retinal dystrophy and corpus callosum abnormalities [16–20]. When analyzing WES data of the proband, we have searched for other patients with *MED13* variants to compare their phenotypes (Table 1) [16–18, 27, 28]. It appears our patient has had the most severe symptoms described so far, raising some doubts in clinicians regarding the diagnosis. The cause of death was not officially established, however, it is notable that the baby’s heart anomalies were more severe compared to the other patients, who had subaortic stenosis, history of murmur (with normal echo and ECG) and dilated aortic root and pulmonary artery [16]. Moreover, bronchopulmonary dysplasia and ileal atresia were never previously reported in patients with MED13-associated syndrome, to our knowledge– those symptoms could be responsible for the lethal outcome. However, bronchopulmonary dysplasia and ileal atresia in newborns are not extremely rare, so the possibility of a coincidence of those clinical symptoms with MED13-associated symptoms remains.

**Table 1:** Clinical characteristic of patients with *MED13 de novo* variants

	Proband	Snijders Blok et al., 2018	Kahrizi et al., 2019	De Nardi et al., 2020	Rogers et al., 2021	Trivisano et al., 2022	Patient: 263,479	Patient: 270,069	Patient: 293,948	Patient: 294,297	Patient: 307,561
							Decipher	Decipher	Decipher	Decipher	Decipher
Variant (NM_005121)	p.Pro835Ser	see [16]	p.Ala443GlufsTer6	p.Pro327Ser	p.Gly1150Glu	p. Tyr834Cys	p.Gln2060lys	p.Thr326Lys	p.Asp1809Glu	p.Asp1368TyrfsTer11	c.2476_2476 + 1​delinsT
Gender	M	see [16]	F	F	M	M	F	M	F	M	M
Age at FU	2 M	see [16]	8 Y	13 Y	7 Y	24 M	NR	NR	NR	NR	NR
Congenital heart abnormalities	+	3/13	NR	-	-	NR	NR	NR	NR	NR	NR
Dysmorphic facial features	+	NR	+	+	+	+	+	+	+	NR	+
Hearing loss	NA	2/13	NR	+	-	+	NR	NR	NR	NR	NR
Eye/vision abnormalities	+	8/13	NR	+	-	+	+	NR	NR	+	NR
Skeletal abnormalities	NA	7/13	NR	+		NR	NR	NR	NR	NR	NR
Growth delay/restriction	+	NR	NR	+	+	+	NR	NR	NR	NR	NR
Hypotonia	+	3/13	NR	+ (infantile)		+	NR	NR	NR	NR	NR
Intellectual developmental delay	NA	13/13	+	+/Mild ID	+/Global DD	+/Severe DD	NR/ Global DD	+/Moderate ID	+/ Global DD	NR/Global DD	+
Delayed motor development	NA	7/13	NR	+	+	+	+	NR	NR	NR/ Global developmental delay	NR
Other	Hypoplastic CC, brain stem atrophy, ileal atresia, bronchopulmonary dysplasia, neonatal death		Microcephaly, long phithrum, prognathism			Epileptic encephalopathy, dysmorphic and hypoplasic CC, posterior enlargement of lateral ventricles		Generalized keratosis follicularis	Joint hypermobility	Joint hypermobility	Impaired pain sensation

FU– follow up; M– male; F– female; CC– corpus callosum; DD– developmental delay; ID– intellectual deficiency; Y– years; M– months; NR– not reported; NA– not assessed

While the same variant, p.Pro327Ser, was reported in two unrelated patients, it is interesting to compare their phenotypic features. Both children presented with intellectual deficiency (described as mild in one of the cases), speech delay, motor developmental delay, conductive hearing loss, scoliosis, and hypotonia (only as infantile form in one of the cases). However, the female patient reported by De Nardi et al. [17] had significant facial dysmorphisms resembling Kabuki syndrome, while no significant facial dysmorphisms were reported in the male patient described by Snijders Blok et al. [16], who had an additional cardio phenotype presented with dilated aortic root and pulmonary artery. This comparison serves as proof for substantial phenotypic variability among the patients with MED13-associated syndrome, even in carriers of the same variant.

The observed p.Pro835Ser missense change has not been described as part of a protein functional center, according to UniProt database [29]. However, the position is highly conserved among 100 vertebrate species, as indicated by the multi alignment available through UCSC genome browser [30], indicating its probable functional importance. Variant effect prediction tools qualify the observed missense as probably damaging (PolyPhen-2: 0.996; Sift: 0.0; CADD: 26.1).

Another missense variant p.Tyr834Cys was recently described as *de novo* in patient with severe developmental delay, epileptic encephalopathy, microcephaly, dysmorphic features and abnormalities of corpus callosum, resembling features observed in the present case (except epilepsy). These two missenses occurred in the neighboring residues, and the produced phenotype in both cases was more severe compared to the previously reported patients. Finally, taking into account all the available evidence, the observed p.Tyr834Cys variant was classified as likely pathogenic according to the ACMG criteria [31].

It is known that the type of the variant can influence the severity of phenotype. For MED13 and MED13L, parts of the Mediator complex, both missense and loss-of-function variants were reported as pathogenic. Notably, missense changes in MED13L were described in association with a more severe phenotype– patients having higher incidence of seizures, MRI abnormalities, autistic features, and cardiac anomalies [26]; thus, we speculated that it might be the same for MED13 protein, and compared clinical features in respect to the variant type (missense vs. loss-of-function). So far, 13 patients carrying *MED13* missense and in-frame amino acid deletion variants were described in the literature [16–20] and Decipher database. Of them, 6 were reported to have mild/borderline intellectual deficiency (ID), 1– moderate ID, 4– global/severe developmental delay (DD), in 2 cases the degree of DD was not assessed but one of them was stated as having severe speech delay. Our patient, being the 14th patient with missense variants, was to young for the assessment. The presence of other features, like seizures, congenital cardiac defects, hypotonia, brain MR abnormalities was also not dependent of variant type.

## Conclusions

In summary, using whole exome trio sequencing, we identified a novel *de novo* likely pathogenic p.Pro835Ser missense variant in *MED13* gene, allowing for genetic diagnosis of a neonate presenting with multiple congenital anomalies and dysmorphisms. The patient had a severe phenotype, that had not been previously reported. This case adds to our knowledge of the recently described MED13-related syndrome and can possibly expand its phenotype.

## Methods

This case study was carried out according to the Code of Ethics of the World Medical Association (Declaration of Helsinki) and the study protocol was reviewed and approved by the Ethics Committee of the Kulakov National Medical Research Center for Obstetrics, Gynecology and Perinatology (Protocol No.7 from Apr 19 2022). Participants (parents) gave written informed consent to the use of any data for scientific purposes and to publish an information concerning their child in open-access online journal. Additionally, the Whole-Exome Newborn Screening Project “EXAMEN” study protocol was reviewed and approved by the Ethics Committee of the Kulakov National Medical Research Center for Obstetrics, Gynecology and Perinatology (Protocol No.9 from Oct 22 2020).

Chromosomal microarray analysis was performed on Affymetrix CytoScan Optima Array. DNA was isolated from the patient’s peripheral lymphocytes. The test revealed no significant aneuploidies, microdeletions or microduplications.

Whole-exome sequencing was performed on an Illumina NovaSeq 6000 instrument with average on-target coverage 89× using xGen™ Exome Hyb Panel v2 (“IDT”, USA) for library preparation. Bioinformatics analysis was performed using an in-house software pipeline designed to detect single-nucleotide variants (SNVs). The variants of interest were manually classified according to the ACMG and AMP guidelines.

**Fig. 3:**
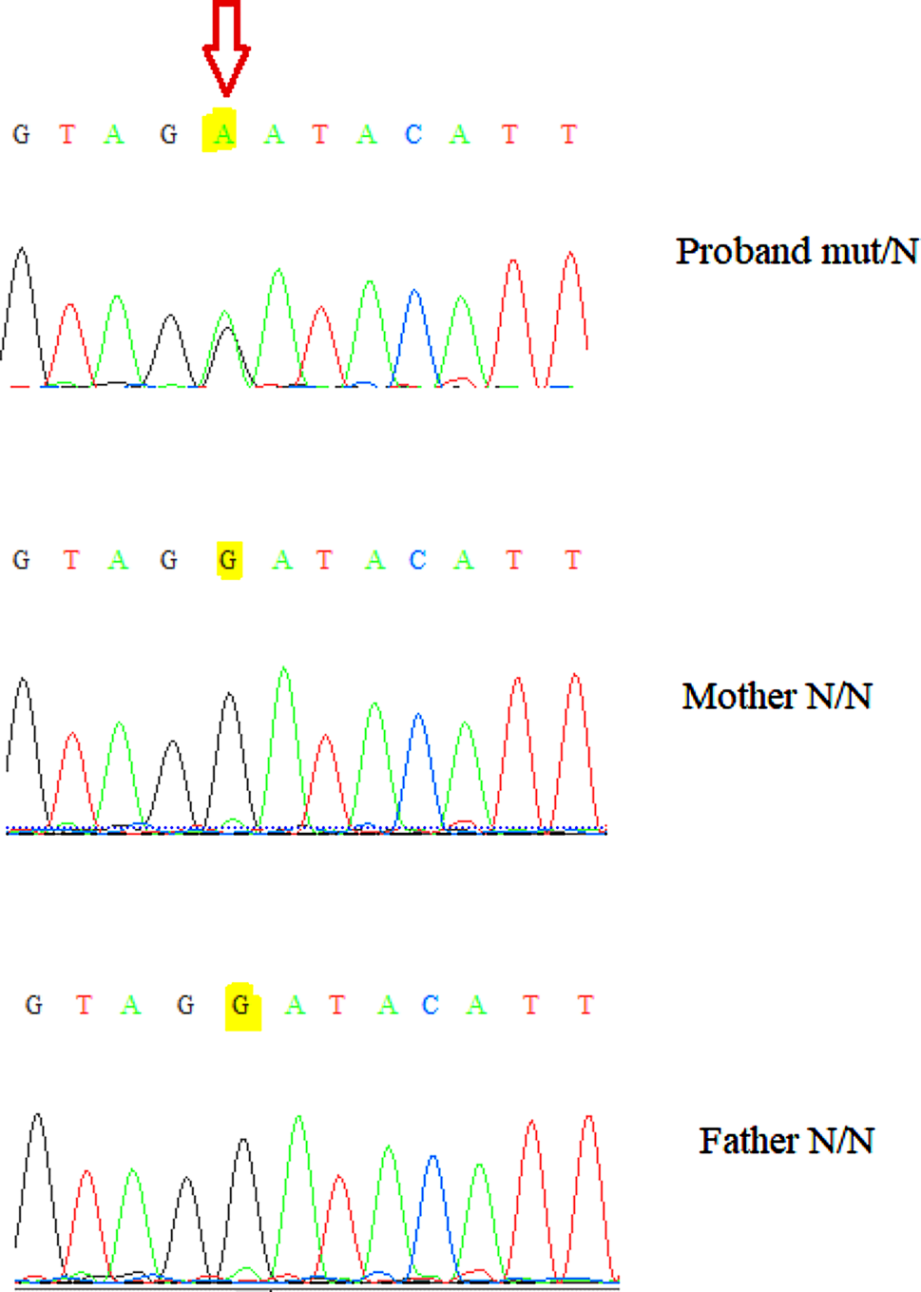
Sanger sequencing presenting the *de novo* origin of the observed p.Pro835Ser variant

A novel *de novo* heterozygous missense variant was detected (c.2503 C > T, NM_005121), predicting p.Pro835Ser missense change in *MED13* gene. Recently, variants in *MED13* were described in patients with congenital anomalies and intellectual developmental disorder (OMIM: 618,009). The variant was absent from the gnomAD population database (v2.1.1, v3.1.2) and has not been previously described in a patient, to our knowledge. Based on the available evidence, taking into account the *de novo* status of the variant, it was classified as likely pathogenic. The *de novo* status of the variant was confirmed by Sanger sequencing of the family trio (parentage verified) using 3730xl DNA Analyzer (Thermo Fisher Scientific, USA) (see Fig. 3). No other relevant SNVs were identified.

## Data Availability

The datasets used and/or analysed during the current study available from the corresponding author on reasonable request.

## Abbreviations

ACMG: The American College of Medical Genetics and Genomics
AMP: Association for Molecular Pathology
CDK: Cyclin-dependent kinase
DNA: Deoxyribonucleic acid
ECG: Electrocardiogram
MED: Mediator complex subunit
NA: Not assessed
NR: Not reported
OMIM: Online Mendelian inheritance in man
RNA: Ribonucleic acid
SNV: Single-nucleotide variant
USA: United States of America
WES: Whole exome sequencing
